# Toward the Controlled Synthesis of Nanostructured Si and SiO_*x*_ Anodes for Li-Ion Batteries via SiO_2_ Magnesiothermic Reduction Reaction

**DOI:** 10.1021/acsaem.4c02836

**Published:** 2025-02-06

**Authors:** Pedro Alonso Sánchez, Kesavan Thangaian, Ole Andreas Øie, Anders Gaarud, Miguel Rodríguez Gomez, Vadim Diadkin, Javier Campo, Federico Hector Cova, María Valeria Blanco

**Affiliations:** † Physics Condensed Matter Department, Aragon Nanoscience and Materials Institute (CSIC - University of Zaragoza), C/Pedro Cerbuna 12, 50009 Zaragoza, Spain; ‡ Department of Materials Science and Engineering, Norwegian University of Science and Technology, Trondheim 7491, Norway; ¶ Swiss-Norwegian Beamline, European Synchrotron Radiation Facility, Avenue des Martyrs 71, 38042 Cedex 9 Cerdanyola, France; § ALBA Synchrotron, Carrer de la Llum 2-26, Cerdanyola del Vallès 08290, Spain

**Keywords:** in situ- synthesis, in situ synchrotron diffraction, reaction mechanism, diatom-SiO_2_, silicon, magnesium, Rietveld analysis, reaction parameters

## Abstract

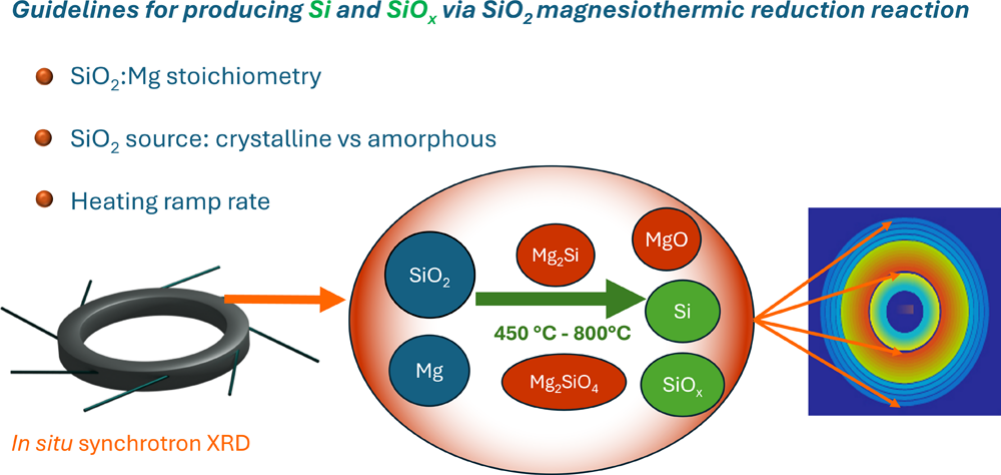

Nanostructured SiOx (0 ≤ *x* ≤ 2) materials are key for boosting energy density in next-generation Li-ion battery anodes, with the magnesiothermic reduction reaction (MgTR) emerging as a scalable pathway for their production from nanoporous SiO_2_. In MgTR, SiO_2_ reacts with Mg at moderate temperatures to form Si and MgO, enabling the preservation of nanostructured features. However, the widespread application of MgTR is hindered by the strong influence of reaction parameters on process dynamics, which leads to the uncontrolled formation of multiple byproducts that not only reduce the Si yield but also require the use of hazardous hydrofluoric acid (HF) for their removal, hampering the synthesis of SiO_*x*_ due to HF’s reactivity with SiO_2_. Hence, a comprehensive understanding of MgTR dynamics and its interplay with reaction parameters constitutes an essential prerequisite toward the effective synthesis of advanced Si and SiO_*x*_ nanostructures. In this work, a systematic approach combining a set of independent time-resolved in situ synchrotron X-ray diffraction studies was employed to provide for the first time a comprehensive understanding of MgTR dynamics under varied reaction conditions, including varied SiO_2_ source (amorphous vs crystalline), different SiO_2_-to-Mg ratios, and different heating ramps. This approach allowed to unveil a complete picture of MgTR and to identify key conditions to prevent byproduct formation. This advancement marks a critical step toward the large-scale zero-carbon footprint synthesis of Si-based anodes for Li-ion batteries, serving as general guidelines for the controlled synthesis of high-purity Si and SiO_*x*_ advanced materials.

## Introduction

The global silicon market is expected to grow from 3.27 million tons in 2024 to 4.25 million tons by 2029,^1^ largely driven by the increasing demand of high energy density Li-ion batteries for electric vehicles, which utilize silicon (Si) and silicon suboxide (SiO_*x*_) negative electrodes.^2−11^ Additionally, the expanding solar photovoltaics industry is contributing to the rise in silicon consumption.^12,13^ Although Si is the second most abundant element in the Earth’s crust, surpassed only by oxygen, and therefore holds great potential for providing cost-effective solutions for technological applications, its conventional production through carbothermal reduction of SiO_2_ raises environmental concerns due to its high energy consumption and significant carbon dioxide emissions.^14,15^ This underscores the need for more sustainable production methods as the demand for Si and SiO_*x*_ continues to rise.

Si production through carbothermal reduction involves the reduction of SiO_2_ with carbon-based sources, such as coal, charcoal, woodchips, or coke,^16^ in an electric arc furnace at temperatures close to 1900 °C to drive the reaction:
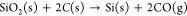


This energy-intensive process requires approximately 157 MWh of energy per ton of Si produced and releases approximately 4.7 kgCO_2_/kgSi directly from the reaction, with additional emissions of 10.5–12 kgCO_2_/kgSi stemming from the electricity used to power the arc furnace.^15^ While transitioning to renewable energy sources for furnace operation may mitigate indirect CO_2_ emissions, addressing the direct emissions inherent to silicon production remains a key challenge.

Metallothermic reduction reactions, including aluminothermic and magnesiothermic reactions, have attracted attention as potential zero-carbon footprint and cost-effective routes for synthesizing Si and SiO_*x*_ materials from naturally abundant SiO_2_ feedstock.^17−22^ Recent advances in magnesiothermic reduction of SiO_2_ (MgTR) have demonstrated its scalability potential, which has boosted the research in this area for the synthesis of Si-based advanced anode materials for Li-ion battery technology.^23−34^ MgTR involves the reduction of SiO_2_ with Mg at moderate temperatures, from 500 to 900 °C, yielding Si and MgO, with the reaction occurring under an inert atmosphere:
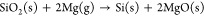
1

A key advantage of the MgTR is its ability to preserve intricate nanostructures during the reduction process, making it particularly attractive for applications that require precise control over material morphology and opening the path for the production of high-performance nanoporous Si-based anodes from nanostructured SiO_2_ templates.^19,24,35^ However, despite these advantages, MgTR faces several challenges, which are mainly related to the high sensitivity of the reaction dynamics with the reaction parameters. MgTR process often yields undesirable byproducts, such as Mg_2_Si, Mg_2_SiO_4_, and MgSiO_3_, which reduce the overall yield and purity of the desired silicon product. Although the origin of these compounds is not clear, they are believed to be formed from side reactions driven by localized heating and magnesium vapor diffusion,^36−39^ and their removal typically requires aggressive chemical treatments, including the use of hazardous hydrofluoric acid (HF), posing significant environmental and safety concerns.^20,40^ The primary side reactions are summarized as follows:
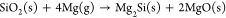
2
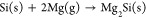
3
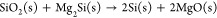
4
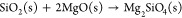
5

While the MgTR process is predominantly utilized for Si production,^41−44^ it has more recently proven effective for synthesizing SiO_*x*_ materials. By adjusting the SiO_2_ to Mg molar ratio, partial reduction of the SiO_2_ template can be achieved,^45,46^ allowing for the controlled synthesis of SiO_*x*_ compounds with varying oxygen content. Importantly, these differences will translate to distinctive functionalities of the material.^47,48^ However, achieving high-purity Si and SiO_*x*_ materials for practical applications requires careful control over reaction conditions to minimize the formation of magnesium silicide and magnesium silicate byproducts. Although MgO and Mg_2_Si can be effectively removed from reacted samples by implementing a simple acid treatment with HCl, the removal of Mg_2_SiO_4_ and MgSiO_3_ impurities requires the use of highly corrosive HF,^20,40^ which presents severe environmental and health hazards and constitutes a significant obstacle toward the widespread adoption of MgTR. Furthermore, the use of HF hampers the production of high-purity SiO_*x*_ compounds through MgTR of SiO_2_, as it also dissolves unreacted SiO_2_ domains.^49^

Although MgTR is being positioned as a highly promising route for producing advanced Si-based nanostructures for battery anodes, its high sensitivity to reaction parameters has driven research to investigate MgTR under various conditions.^24,36−39,50−52^ However, the lack of a systematic and comprehensive approach to track in real time temperature-dependent reaction dynamics of MgTR when using different SiO_2_ precursors, different temperature heating ramps, and different SiO_2_ to Mg ratios hinders the development of rational strategies to fully control MgTR dynamics, which constitutes an essential prerequisite toward the synthesis of advanced Si-based nanostructured materials.

This study presents, for the first time, a systematic investigation of the SiO_2_ MgTR mechanism under varying conditions using time-resolved in situ synchrotron X-ray diffraction coupled with Rietveld analysis. The aim is to provide a comprehensive understanding of MgTR, facilitating the controlled synthesis of advanced Si-based materials. A series of independent in situ MgTR experiments were conducted using both nanostructured crystalline and amorphous SiO_2_ precursors, which were mixed at different SiO_2_:Mg molar ratios and heated at varying temperature ramps. This approach allowed for the exploration of the influence of the SiO_2_ source, reactant stoichiometry, and heating protocol on MgTR dynamics. The resulting analysis offers an unprecedented, thorough examination of the MgTR process, enabling the establishment of general guidelines for optimizing MgTR and providing strategies to enhance the yield and purity of Si and SiO_*x*_ materials for sustainable technological applications.

## Results and Discussion

**Figure 1:**
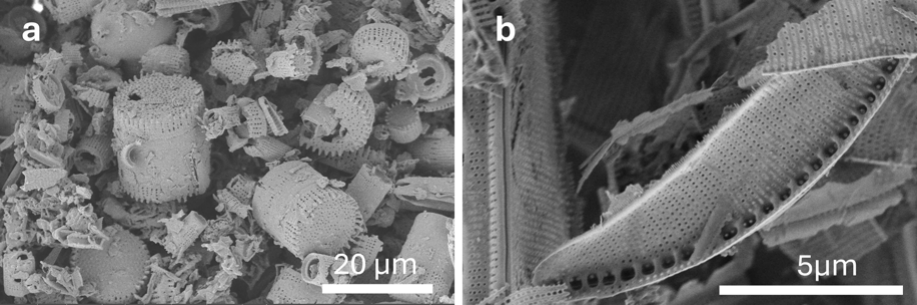
SEM micrographs of (a) nanostructured SiO_2_ particles from diatomaceous earth (DE-SiO_2_) and (b) nanostructured SiO_2_ particles from single-species industrially cultured Nitzschia sp. diatoms (NZ-SiO_2_).

This section presents first a systematic and comparative analysis of the impact of 3 different SiO_2_ to Mg molar ratios on the MgTR dynamics of powder mixtures containing crystalline SiO_2_ from diatomaceous earth (DE) and amorphous SiO_2_ from the shells of Nitzschia sp. diatoms (NZ) as reagents. Then, a similar analysis is provided to assess the effect of the heating ramp on the MgTR process. Figure 1 presents SEM micrographs showing the morphological differences of crystalline DE-SiO_2_ and amorphous SiO_2_ from cultured diatoms. A detailed description of the properties of both SiO_2_ precursors has been already reported by the authors.^53^

### Effect of SiO_2_:Mg Molar Ratios and SiO_2_ Source

The results of the in situ XRD MgTR of samples containing SiO_2_:Mg:NaCl molar ratios of 1:2:2.5 (DE-1 and NZ-1), 1:1.5:2.5 (DE-2 and NZ-2), and 1:1:2.5 (DE-3 and NZ-3) are presented according to the nomenclature introduced in Table 4.

#### SiO_2_:Mg:NaCl 1:2:2.5, DE-SiO_2_ vs NZ-SiO_2_

**Figure 2:**
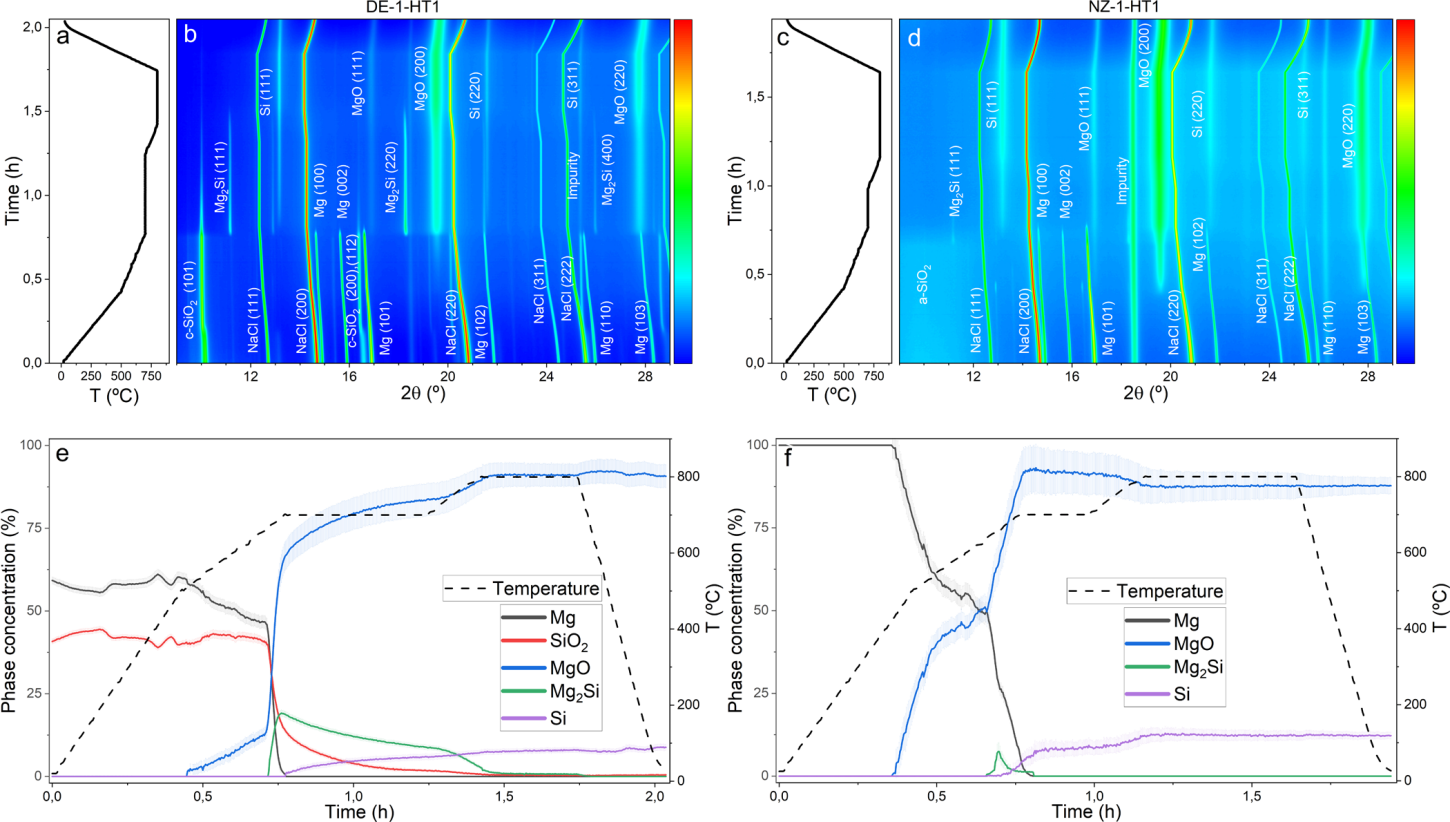
Temperature evolution during in situ MgTR experiments of (a) DE-1-HT1 and (c) NZ-1-HT1. Contour plot showing the corresponding evolution of the X-ray diffraction patterns of (b) DE-1-HT1 and (d) NZ-1-HT1. Time- and temperature-dependent evolution of crystalline phase concentrations for experiments: (e) DE-1-HT1 and (f) NZ-1-HT1.

The results corresponding to the DE-1-HT1 and NZ-1-HT1 experiments are displayed in Figure 2. The temperature profiles over the reaction time for each experiment are depicted in Figure 2a,c. On the right side of the figures are displayed contour plots showing the corresponding evolution of the in XRD patterns, Figure 2b,d, respectively. The main Bragg reflections of the crystalline phases present at each temperature are identified within the contour plots. Figure 2d also shows the presence of a Bragg reflection labeled as ‘spurious’. This reflection belongs to an external component located outside the furnace that is not in contact with the sample. Figure 2e,f displays the results of the evolution of crystalline phase percentages as functions of both the experiment time and the temperature.

As shown in Figure 2b, the only phases present at room temperature are SiO_2_, Mg, and NaCl. As the temperature increases, all three phases exhibit a shift in their diffraction peaks toward lower angles, indicative of thermal expansion of the respective crystalline lattices. No significant changes are observed below 513 °C, although small fluctuations in relative amounts of the Mg and SiO_2_ phases are evidenced, Figure 2e, which are accompanied by changes in the peak shapes of DE-SiO_2_. Given that DE-SiO_2_ presents an Opal-C structure similar to cristobalite but lacking of long-range order and containing varying amounts of structural water,^54,55^ such oscillations can be attributed to the release of water trapped within the DE-SiO_2_ structure. At temperatures above 513 °C, a slight emergence of peaks belonging to the MgO phase can be observed, and further heating to 662 °C results in an increased amount of MgO that reaches 15%. At this temperature, the reflections corresponding to Mg and SiO_2_ begin to diminish, as shown in Figure 2e. This observation indicates that MgTR proceeds via a solid–solid reaction mechanism according to reaction 1. However, the formation of crystalline Si (c-Si) is not evidenced, suggesting that the silicon produced in this initial stage would be amorphous Si (a-Si).

Once the temperature exceeds 662 °C, a marked shift in reaction dynamics is clearly observed and attributed to Mg reaching its melting point. At this stage, an equilibrium is established between the evaporation of Mg, driven by its high vapor pressure, and the gas–solid MgTR. This transition from solid–solid to gas–solid reaction regime significantly accelerates the reaction rates, as evidenced by the rapid consumption of SiO_2_ reactant. The progression of SiO_2_ phase evolution can be used as a key indicator of the degree of advancement of the reaction, given that the disappearance of Mg reflections may result from either its melting and evaporation or its conversion to MgO. Upon reaching the melting point of Mg, a rapid emergence of Mg_2_Si reflections is observed. This phase quickly reaches a concentration of 19% at temperatures approaching 700 °C. Notably, this is followed by the growth of c-Si diffraction peaks, concurrent with the decline of Mg_2_Si until its total disappearance from the system. Therefore, the reaction pathway during the gas–solid regime can be summarized as follows: After Mg melts, reaction 2 initially dominates the chemistry of the system, leading to the formation of Mg_2_Si, which then serves as an intermediate for the production of c-Si through reactions 1 and 4. By the end of the experiment, the c-Si content is quantified at 7.8%, with no evidence of silicate formation even at elevated temperatures. It is important to note that an intensity bump, attributed to a-Si, persists at high temperatures until the experiment is concluded. This suggests that the total amount of Si present in the system is significantly larger than the c-Si percentage quantified by the Rietveld analysis.

Figure 2d presents a contour plot corresponding to the NZ-1-HT1 experiment in which amorphous SiO_2_ is used as a reactant. A comparison of Figure 2b,d reveals an intensity halo corresponding to amorphous SiO_2_ reflections at the beginning of the NZ-1-HT1 experiment. Additionally, significantly broader peaks are observed for the phases formed at high temperatures, such as Si, Mg_2_Si, and MgO, indicating the formation of smaller crystallites and higher internal stresses in this sample compared to those in the DE-1-HT1 experiment. Figure 2f shows the corresponding phase composition changes. No phase transitions occur below 413 °C. At this temperature, reflections of MgO start to emerge, indicating that the reduction of SiO_2_ begins approximately 100 °C lower than in the DE-1-HT1 experiment, which used crystalline SiO_2_. Analysis of the phase concentration curves reveals two distinct temperature-driven processes leading to MgO formation, similar to those observed in DE-1-HT1. Up to 630 °C, the solid–solid MgTR proceeds at a steady but slow rate, with the MgO concentration reaching about 50% of the total crystalline phases. During this stage, no new crystalline phases are detected, consistent with the observations from the DE-1-HT1 experiment.

As the system approaches temperatures near the melting point of Mg, peaks belonging to Mg_2_Si begin to appear and a sharp increase in the slope of the MgO concentration curve is observed. The simultaneous increase in Mg_2_Si concentration and decrease in SiO_2_ content clearly indicate that the reaction rate of reaction 2 significantly accelerates when Mg melts. With further temperature increases, the amount of Mg_2_Si in the sample begins to decline, suggesting that at higher temperatures, the reaction rate of reaction 4 surpasses that of reaction 2. This eliminates the necessity of Mg_2_Si as an intermediate phase for the MgTR. An additional finding is the difference in the slopes of MgO and Mg_2_Si phase evolution curves between Figure 2e,f. In the first case, during the solid–solid regime, the MgO phase increment follows a linear slope, whereas in the second case, this linear increase is followed by a stage where the slope markedly decreases. This stage, where the formation rate of MgO slows, marks the onset of Mg_2_Si formation. Additionally, the Mg_2_Si phase coexists with MgO for a shorter period in the NZ-1-HT1 experiment compared to the DE-1-HT1 case, indicating a faster transition through this intermediate phase.

#### SiO_2_:Mg:NaCl 1:1.5:2.5, DE-SiO_2_ vs NZ-SiO_2_

**Figure 3:**
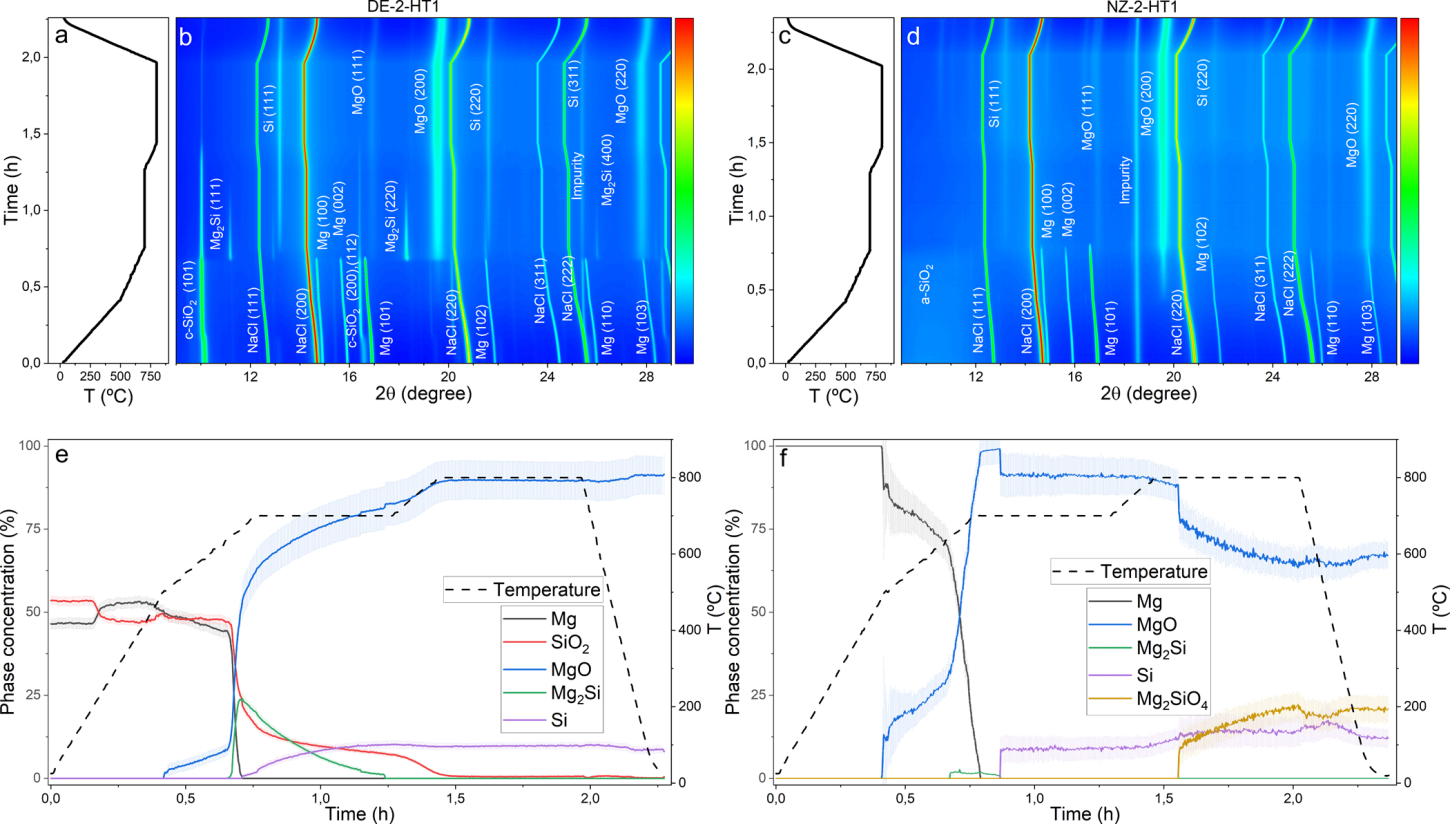
Temperature evolution during in situ MgTR experiments (a) DE-2-HT1 and (c) NZ-2-HT1. Contour plot showing the corresponding evolution of the X-ray diffraction patterns of (b) DE-2-HT1 and (d) NZ-2-HT1. Time- and temperature-dependent evolution of crystalline phase concentrations for experiments (e) DE-2-HT1 and (f) NZ-2-HT1.

Figure 3 shows in situ results corresponding to experiments DE-2-HT1, Figure 3a,b, and NZ-2-HT1, Figure 3c,d. Again, SiO_2_, Mg, and NaCl appear as the only phases present at room temperature. Both contour plots exhibit intensity halos at high temperatures, which are indicative of the formation of amorphous phases as previously evidenced in the MgTR of samples having 1:2:2.5 stoichiometries.

The evolution of phase percentages during the DE-2-HT1MgTR experiment is displayed in Figure 3e. Similar to the DE-1-HT1 case, the MgTR proceeds sequentially, with a solid–solid reaction occurring at temperatures below the Mg melting point, followed by a gas–solid reaction at higher temperatures. This transition in reaction regime is again marked by a significant increase in reaction kinetics, leading to faster reaction rates. MgO formation initiates at around 500 °C, which is similar to previous observations in the DE-1-HT1 experiment. Again, Mg_2_Si appears as an intermediate phase, initially coexisting with c-SiO_2_, Mg, and MgO. The Mg_2_Si content reaches a maximum value of 24% at 670 °C, after which it reacts with c-SiO_2_ to form c-Si and MgO, in accordance with reaction 4. It is also evidenced that c-SiO_2_ does not completely disappear even at 700 °C, and traces of this phase persist up to 800 °C. By the end of the experiment, c-Si, MgO, NaCl, and a minor amount of SiO_2_ are the only phases detected. The residual c-SiO_2_ was expected due to the excess of c-SiO_2_ used in the reactants mixture; however, the amount of c-SiO_2_ detected does not match the stoichimetry of the reaction. This discrepancy suggests that some of the SiO_2_ may have partially decomposed without reacting, losing its long-range order. Additionally, the final c-Si content is measured at 9.5%, which further supports the presence of a-Si among the reaction products.

The MgTR dynamics observed in the NZ-2-HT1 experiments shows an early temperature onset of MgO formation, starting at 480 °C and reaching a maximum concentration of 28% when the reduction reaction proceeds under solid–solid regime (Figure 3f). Similar to the previous section, only small amounts of Mg_2_Si phase are formed (below 2%) compared to DE-2-HT1 MgTR experiment, and this phase exists for a very short time period. A key difference, however, is the formation of forsterite, Mg_2_SiO_4_, when the temperature reaches 800 °C, indicating the occurrence of reaction 5. This phase accounts for around 20% of the total crystalline phases at the end of the reaction. The formation of Mg_2_SiO_4_ during MgTR is not fully understood in the literature and has been primarily attributed to three factors: reaction time, high reaction temperatures, and Mg deficiency. A comparison of the DE-2-HT1 and NZ-2-HT1 experiments reveals that MgTR proceeds more rapidly when amorphous SiO_2_ is used as a reactant. This suggests that the additional temperature and reaction time after the completion of MgTR promote the reaction between the remaining SiO_2_ and MgO to form Mg_2_SiO_4_. Consequently, lowering the reaction temperature and shortening the reaction time may be effective strategies for synthesizing pure SiO_*x*_ materials through MgTR.

#### SiO_2_:Mg:NaCl 1:1:2.5, DE-SiO_2_ vs NZ-SiO_2_

**Figure 4:**
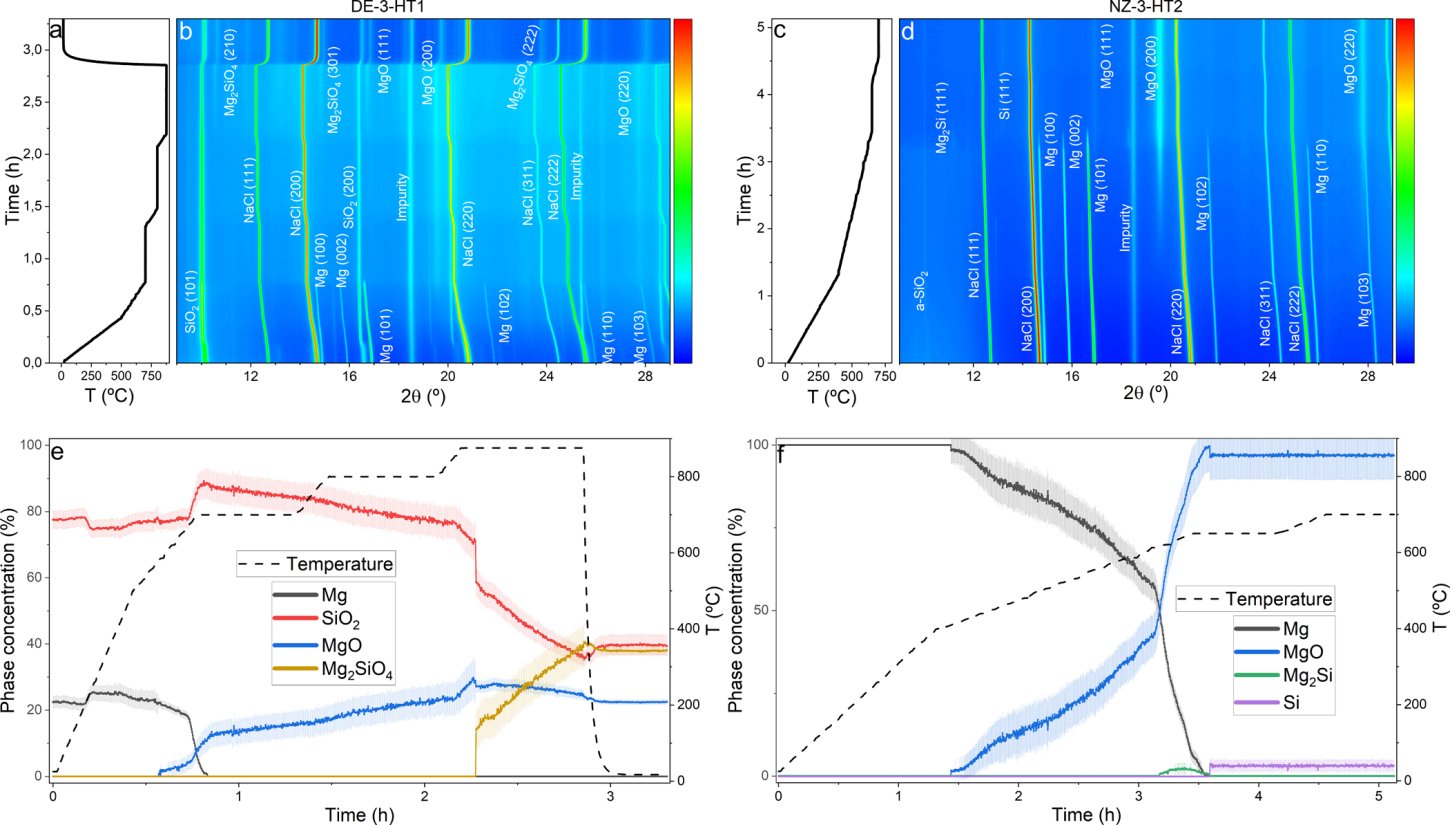
Temperature evolution during in situ MgTR experiments of (a) DE-3-HT1 and (c) NZ-3-HT2. Contour plot showing the corresponding evolution of the X-ray diffraction patterns of (b) DE-3-HT1 and (d) NZ-3-HT2. Time- and temperature-dependent evolution of crystalline phase concentrations for experiments (e) DE-3-HT1 and (f) NZ-3-HT2.

The results of the MgTR experiments for samples with the lowest Mg content, corresponding to the DE-3-HT1 and NZ-3-HT2 runs, are presented in Figure 4a,b,e and c,d,f, respectively. In the DE-3-HT1 experiment, an additional heating step up to 875 °C was introduced to promote the formation of Mg_2_SiO_4_ through reaction 5. From the phase evolution data in Figure 4e, it is evident that both the temperature and holding time significantly influence the final amount of Mg_2_SiO_4_ in the products. A 45 min holding step at 875 °C increased the amount of Mg_2_SiO_4_ from 20 to 40%. In contrast, the NZ-3-HT2 experiment was conducted up to 700 °C to determine whether the formation of the Mg_2_SiO_4_ phase could be suppressed by using amorphous silica and carefully controlling the reaction temperature.

As depicted in Figure 4e, the MgO formation mechanism in DE-3-HT1 is similar to that observed in previous experiments. The MgO formation initiates at 560 °C and accelerates upon reaching the Mg melting point. However, due to the reduced Mg content in this experiment, neither Mg_2_Si as an intermediate nor c-Si is detected. Instead, the presence of an intensity halo at high reaction temperatures, along with the consumption of c-SiO_2_ and the increase of the MgO phase, suggests the formation of a-Si. At 875 °C, this intensity halo becomes more pronounced, and the formation of Mg_2_SiO_4_ is clearly evident. By the end of the experiment, the relative amounts of MgO, SiO_2_, and Mg_2_SiO_4_ crystalline phases are 22, 38, and 40%, respectively. A comparison of results from DE-1-HT1, DE-2-HT1, and DE-3-HT1 samples indicates that the formation of Mg_2_Si increases with higher Mg content in the reactant mixture.

For the NZ-3-HT2 experiment, which was conducted using a slower temperature ramp, MgO formation begins at 400 °C. Again, the reaction rate increases significantly upon reaching 650 °C. However, the formation of c-Si remains minimal, not exceeding 3%, as shown in Figure 4f, indicating that most of the Si is formed as a-Si. An important finding from this experiment is that maintaining the reaction temperature at 700 °C effectively prevents Mg_2_SiO_4_ formation. Additionally, the absence of Mg_2_Si formation in this experiment reinforces the trend observed with crystalline SiO_2_ as the reactant: Mg_2_Si formation does not occur when the Mg content is low and increases with a higher Mg content in the reactant mixture.

**Figure 5:**
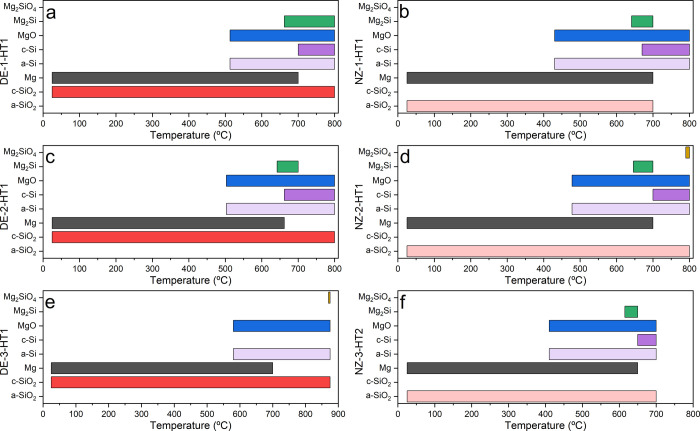
Temperature ranges of observed crystalline phases during experiments: (a) DE-1-HT1, (b) NZ-1-HT1, (c) DE-2-HT1, (d) NZ-2-HT1, (e) DE-3-HT1, and (f) NZ-3-HT2.

The dynamics of the MgTR reaction mechanism using crystalline and amorphous silica as reagents can be summarized as follows. The reaction primarily proceeds via reaction 1, with a notable kinetic advantage when amorphous SiO_2_ is used as reactant. This is evidenced by a lower onset temperature for MgO formation and a higher rate of Mg consumption under the solid–solid SiO_2_ regime. Across all experiments, a significant acceleration in the reaction rates occurs upon reaching the Mg melting temperature; at this point, the reaction transitions to a gas–solid regime. The Mg_2_Si emerges as an intermediate phase for SiO_2_ to Mg ratio above 1:1.5, with its quantity increasing as the ratio rises to 1:2. This phase is more prominent in samples containing crystalline SiO_2_. The formation of Mg_2_SiO_4_ was observed at temperatures above 800 °C for NZ samples and above 875 °C for DE samples. Lowering the reaction temperature proved to be an effective approach to preventing Mg_2_SiO_4_ formation. A summary of the phases detected at different temperatures for each experiment is provided in Table 1, with the corresponding ranges for phase detection depicted in Figure 5. Also, a summary of the sample composition at the end of each experiment is provided in Table 2.

**Table 1:** Summary of Temperature Reactions

	a-SiO_2_	c-SiO_2_	Mg	a-Si	c-Si	MgO	Mg_2_Si (°C)	Mg_2_SiO_4_
DE-1-HT1		RT-800 °C	RT-700 °C	513 °C-end	700 °C-end	513 °C-end	662–800	
NZ-1-HT1	RT-700 °C		RT-700 °C	430 °C-end	670 °C-end	430 °C-end	641–700	
DE-2-HT1		RT-end	RT-662 °C	503 °C-end	662 °C-end	503 °C-end	642–700	
NZ-2-HT1	RT-end		RT-700 °C	478 °C-end	700 °C-end	478 °C-end	646–700	800 °C-end
DE-3-HT1		RT-end	RT-700 °C	580 °C-end		580 °C-end		875 °C-end
NZ-3-HT2	RT-end		RT-650 °C	410°-end	650 °C-end	410 °C-end	615–650	

**Table 2:** Final Products for Each Experiment

	weight fraction (%)						molar fraction (%)					
	Mg	c-SiO_2_	MgO	Mg_2_Si	c-Si	Mg_2_SiO_4_	Mg (mol)	SiO_2_ (mol)	MgO	Mg_2_Si	c-Si	Mg_2_SiO_4_
DE-1-HT1	0	0	91	0	9	0	0.00	0.00	87.57	0.00	12.43	0.00
DE-2-HT1	0	0	91	0	9	0	0.00	0.00	87.57	0.00	12.43	0.00
DE-3-HT1	0	40	21	0	0	39	0.00	45.47	35.59	0.00	0.00	18.94
NZ-1-HT1	0	0	87	0	12	0	0.00	0.00	83.48	0.00	16.52	0.00
NZ-2-HT1	0	0	67	0	12	21	0.00	0.00	74.25	0.00	19.08	6.67
NZ-3-HT2	0	0	97	0	3	0	0.00	0.00	95.75	0.00	4.25	0.00

**Figure 6:**
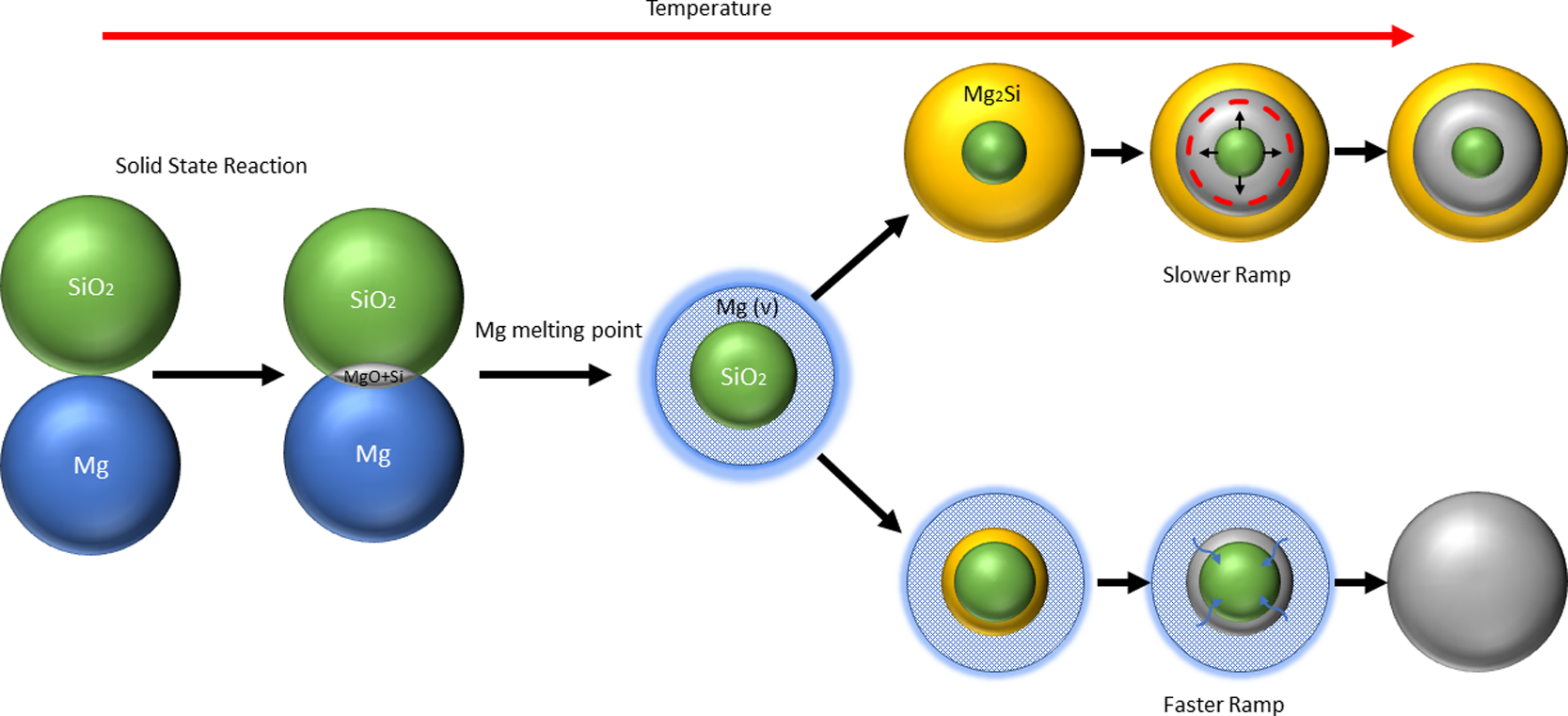
Proposed phase evolution paths for SiO_2_ MgTR of samples heated at different heating ramps.

A general trend observed across all experiments is that the reaction kinetics are faster in samples with amorphous SiO_2_ compared to those with crystalline SiO_2_, which can be attributed to the larger surface area per unit volume of amorphous SiO_2_, which provides more nucleation sites and larger diffusion channels, allowing Mg to penetrate the SiO_2_ structure more efficiently, thus making the reaction more favorable.

### Effect of Heating Ramp Rate

The influence of the temperature ramp during the MgTR was investigated. To this end, several experiments were performed on powder mixtures with a SiO_2_:Mg:NaCl molar ratio of 1:2:2.5, a stoichiometry that allows for complete reduction of silica. The DE-1 sample was subjected to 3 different heating protocols, which involved heating ramps of 10, 50, and 100 °C/min, which correspond to experiments IDs DE-1-HT3, DE-1-HT4, and DE-1-HT5 from Table 4, respectively. In each case, the samples were heated continuously from room temperature to 875 °C without any temperature holding steps.

**Figure 7:**
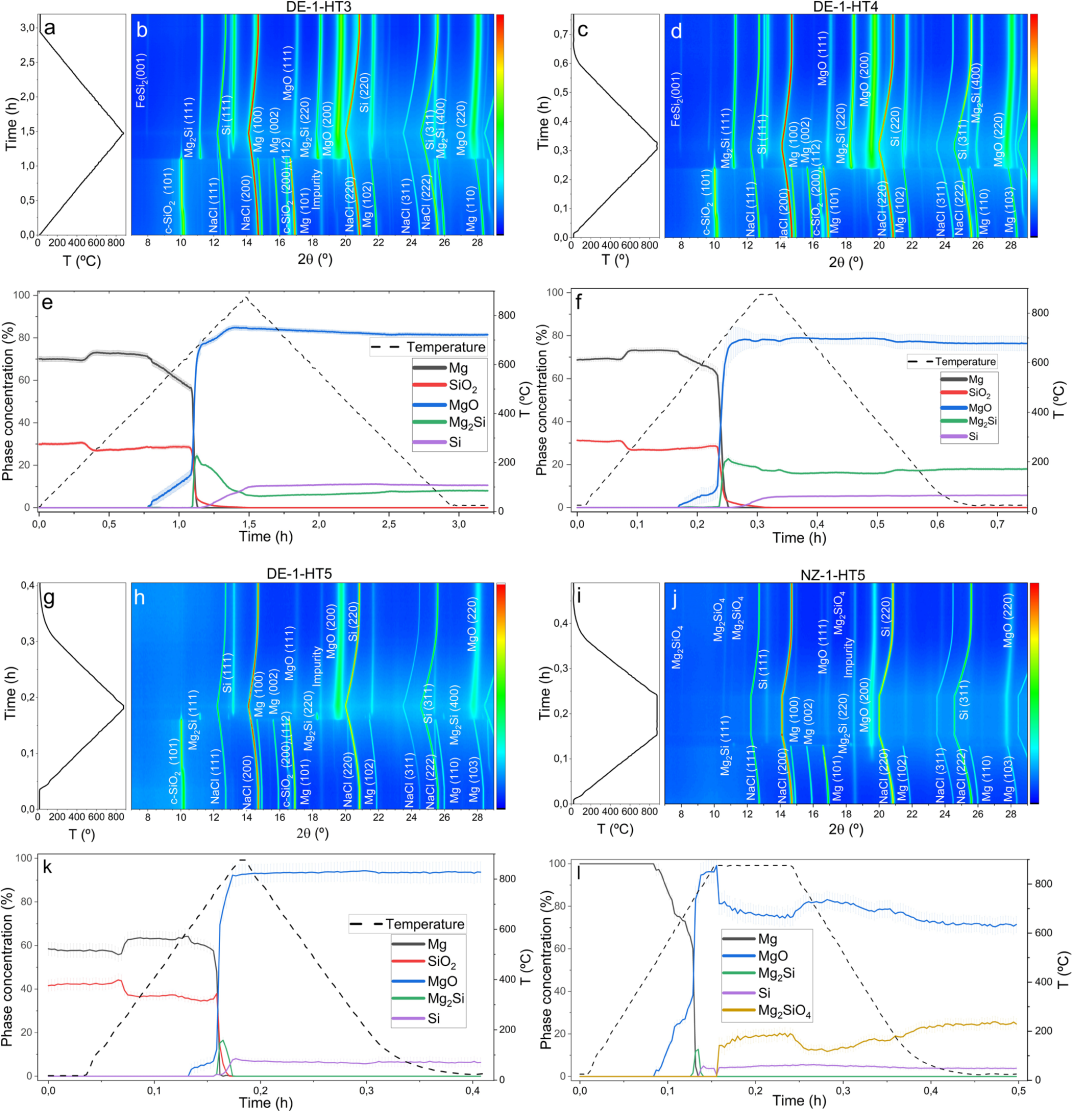
Temperature evolution during in situ MgTR experiments of (a) DE-1-HT3, (c) DE-1-HT4, (g) DE-1-HT5, and (i) NZ-1-HT5. Contour plot showing the corresponding evolution of the XRD patterns of (b) DE-1-HT3, (d) DE-1-HT4, (h) DE-1-HT5, and (j) NZ-1-HT5. Time- and temperature-dependent evolution of crystalline phase concentrations for experiments (e) DE-1-HT3, (f) DE-1-HT4, (k) DE-1-HT5, and (l) NZ-1-HT5.

The results corresponding to heating ramps of 10, 50, and 100 °C/min are displayed in Figure 7a,c,g, respectively and include graphs of temperature evolution, XRD contour plots, and phase quantification results from Rietveld analysis. Across all heating rates, the MgTR mechanism exhibits similar phase evolution dynamics to those previously described. Again, MgO formation occurs when the reaction is under both solid–solid and gas–solid regime. Additionally, the formation of FeSi_2_ is detected at high temperatures (875 °C). One of the impurities in diatomaceous earth is iron, which explains the formation of this silicide. The main Bragg reflections of FeSi_2_ are indicated within the contour plots. However, the amount of this phase remains below 1% and has therefore been excluded from the sequential Rietveld analysis.

The most important difference observed between the three temperature heating ramps is the distinct evolution of the Mg_2_Si phase. For experiments DE-1-HT3 and DE-1-HT4, corresponding to heating ramps of 10 and 50 °C/min, respectively, Mg_2_Si persists as a final product at the end of the reaction. In these experiments, Mg_2_Si forms rapidly right after the melting point of Mg is reached, achieving concentration values of 24, 21, and 15% (HT3, HT4, and HT5, respectively). Following this, Si appears according to eq 4. Despite this, the final amount of Mg_2_Si is 5 and 15% for HT3 and HT4, respectively, indicating incomplete conversion to Si for the lower temperature ramps.

The findings underscore the complex interplay between the maximum temperature and the temperature ramp. Specifically, at a heating ramp of 10 °C/min, the presence of an intermediate holding step at a lower temperature leads to the complete consumption of Mg_2_Si (Figure 2e). In contrast, without such a holding step, as depicted in Figure 7e, Mg_2_Si is not fully consumed. To achieve complete conversion of Mg_2_Si, a higher heating rate of 100 °C/min is required.

A possible explanation for the observed discrepancies at different heating rates is illustrated in Figure 6. Initially, the solid–solid reaction would occur solely at the interface where SiO_2_ and Mg are in contact. However, once the melting point of Mg is reached and its vapor pressure increases, Mg can fully surround the SiO_2_ particles, creating multiple interfaces along their surfaces. It is at these regions that Mg_2_Si is formed. As demonstrated in previous sections, Mg_2_Si forms within a limited temperature range before decomposing according to reaction 4. Consequently, the heating rate plays a crucial role in determining the amount of Mg_2_Si produced. At lower heating rates, the system has sufficient time to fully convert Mg into Mg_2_Si according to reaction 3 before reaching the decomposition temperature. This results in a core of SiO_2_ surrounded by a shell of Mg_2_Si, as shown in the upper pathway of Figure 6. Once the decomposition temperature is attained, the SiO_2_ core begins to react with the Mg_2_Si shell to form MgO and Si according to reaction 4. However, as the core–shell reaction progresses, the contact surface between Mg_2_Si and SiO_2_ decreases and the reaction is stopped.

For faster temperature ramps, the system follows the lower pathway, as depicted in Figure 6. As illustrated, a smaller amount of Mg_2_Si is formed, resulting in a thinner shell, and not all of the Mg is consumed. Consequently, once the Mg_2_Si decomposition temperature is reached, the reaction between the Mg_2_Si and the core proceeds similar to the reaction observed at lower heating rates. However, a thinner MgO and Si allows Mg vapors to diffuse more readily through it. This enhance diffusion enables the system to continue evolving according to reaction 1.

To assess the impact of high temperature heating ramps when using amorphous silica reagent, the HT5 heating cycle was repeated using the NZ-1 sample. This time, the formation of Mg_2_SiO_4_ was observed once the maximum temperature was reached. In contrast, no evidence of silicates was observed in the DE-1-HT5 experiment, similar to the results shown in Figure 3. These findings suggest that the formation of forsterite is more favorable when using amorphous silica compared with crystalline silica.

## Conclusions

This study offers novel insights into the SiO_2_ MgTR mechanism for synthesizing Si and SiO_*x*_ by systematically investigating the effects of SiO_2_ source (amorphous vs crystalline), SiO_2_ to Mg molar ratio (1:1, 1:1.5, and 1:2), and heating ramp rates (10–100 °C/min) using time-resolved in situ synchrotron X-ray diffraction.

The MgTR mechanism can be summarized as follows. Below the Mg melting point, the MgTR proceeds via a solid–solid regime, forming MgO and amorphous Si. The onset temperature is 513 °C when using crystalline SiO_2_ and drops by 100 °C when using amorphous SiO_2_. At the Mg melting point, a gas–solid regime becomes dominant, and rapid formation of Mg_2_Si intermediate phase is evidenced before the formation of crystalline Si. The formation of Mg_2_Si is more favorable when a crystalline SiO_2_ precursor is used, and the Mg_2_Si percentage increases with higher Mg content in the reactant mixture.

MgTR is favored when using an amorphous SiO_2_ precursor. Precursor crystallinity has also been found to affect the formation of Mg_2_SiO_4_, which is formed at 800 °C when using the amorphous SiO_2_ reagent and at 870 °C when using crystalline SiO_2_. Increasing the temperature and reaction time after the MgTR completion promotes the reaction between the remaining SiO_2_ and MgO to form Mg_2_SiO_4_. Hence, lowering the reaction temperature and shortening the reaction time can be effective strategies for synthesizing high-purity SiO_*x*_ materials through MgTR. Also, the Mg_2_SiO_4_ reaction product is evidenced when the Mg amount is decreased in the reagent mixtures.

Regarding the effect of heating ramps, slow ramps result in a higher proportion of the intermediate Mg_2_Si phase, limiting the formation of Si and MgO and leading to higher amounts of Mg_2_Si within reaction products. This limitation can be overcome by applying faster heating ramp rates. The findings of this work shed light on the influence of critical reaction parameters on the dynamic pathways of MgTR and will serve as guidelines for the controlled synthesis of high-purity Si and SiO_*x*_ materials for technological applications.

## Experimental Section

Crystalline nanostructured SiO_2_ from commercially available Diatomaceous Earth (DE, Sigma-Aldrich) and amorphous nanostructured SiO_2_ frustules from industrially cultured Nitzchia sp. diatoms (NZ, Swedish Algae Factory, Sweden) were used as SiO_2_ sources. Both materials were characterized in a previous work by the authors.^53,56,57^ Scanning electron microscopy micrographs of the SiO_2_ reagents were acquired using an FEI SEM Apreo. DE-SiO_2_ and NZ-SiO_2_ were first mixed with Mg powder (≥99%, Sigma-Aldrich) and NaCl (≥99%, Sigma-Aldrich) in molar ratios of 1:1:2.5, 1:1.5:2.5, and 1:2:2.5, respectively, and then subjected to manual grinding for 15 min using an agate mortar and pestle. Each final batch of the powder mixture was 4 g.

Samples were transferred to an Ar-filled glovebox and then loaded into 0.6 mm inner diameter sapphire capillaries. Standard quartz capillaries were avoided to prevent any reaction with Mg from the mixture. Capillaries were sealed inside the glovebox with a ceramic paste to ensure the preservation of inert conditions throughout the experiments. The sealed capillaries were then placed horizontally into a goniometer head, which was connected to a spinning stage that allowed continuous rotation of the sample between 0 and 180°. The samples were subjected to different heat treatments in a horizontal furnace available on the beamline,^58^ which was previously calibrated within the temperature range from 25 to 900 °C by heating a Pt standard and performing automatic calculations of the crystal lattice parameters. The experimental setup is shown in Figure S1 of the Supporting Information (SI).

In situ synchrotron X-ray diffraction experiments were performed to investigate the effects of the SiO_2_ source, heating ramp rate, and temperature on the reaction mechanism of the MgTR. Standard heating protocols involved a sequence of heating steps followed by constant temperature steps at selected temperatures to provide enough time to achieve reaction completion. The influence of the heating ramp rate on MgTR was evaluated by continuously heating the reactant mixture from room temperature to 875 °C, at ramp rates of 10, 50, and 100 °C/min. Details of heating protocols and experimental details are summarized in Tables 3 and 4, respectively.

**Table 3:** Summary of Heat Treatment Protocols for the MgTR and Their Corresponding IDs

heating ID	SiO_2_ heating protocol
HT1	20 °C/min to 500 °C → 10 °C/min to 700 °C → 30 min hold → 10 °C/min to 800 °C → 30 min hold → 50 °C/min to 25 °C
HT2	5 °C/min to 400 °C → 2 °C/min to 650 °C → 30 min hold → 2 °C/min to 700 °C → 30 min hold → 50 °C/min to 25 °C
HT3	10 °C/min to 900 °C
HT4	50 °C/min to 900 °C
HT5	100 °C/min to 900 °C

**Table 4:** Summary of Experiment Name, SiO_2_ Source, Reactants Molar Ratio, and Heating Process for the Different In Situ XRD Experiments

experiment ID	SiO_2_ source	SiO_2_:Mg:NaCl	ramp
DE-1-HT1	diatomaceous earth	1:2:2.5	HT1
DE-1-HT3	diatomaceous earth	1:2:2.5	HT3
DE-1-HT4	diatomaceous earth	1:2:2.5	HT4
DE-1-HT5	diatomaceous earth	1:2:2.5	HT5
DE-2-HT1	diatomaceous earth	1:1.5:2.5	HT1
DE-3-HT1	diatomaceous earth	1:1:2.5	HT1
DE-3-HT5	diatomaceous earth	1:1:2.5	HT5
NZ-1-HT1	Nitzschia sp.	1:2:2.5	HT1
NZ-1-HT5	Nitzschia sp.	1:2:2.5	HT5
NZ-2-HT1	Nitzschia sp.	1:1.5:2.5	HT1
NZ-3-HT2	Nitzschia sp.	1:1:2.5	HT2

Synchrotron powder X-ray diffraction data were collected at the BM01 station of the Swiss-Norwegian beamline (SNBL) of the European Synchrotron Radiation Facility (ESRF). The raw data can be found at ESRF data portal.^59^ A beam with a size of 300 × 3000 μm^2^ and a wavelength of 0.720 Å was used. The diffraction rings were acquired with a Dectris Pilatus 2 M direct photon counting area detector. Frames were collected every 10 s, and the exposure time was 5 s. Calibration was performed using LaB_6_ powder calibrant available at the beamline. The XRD data were integrated using the Dioptas software,^60^ and sequential Rietveld refinement was performed using the Fullprof-based software FullProfApp.^61^ Since NaCl did not react during the MgTR, acting solely as a temperature buffer agent, its phase concentration was excluded from weight percent calculations. Rietveld refinements of selected diffraction patterns can be found in Figures S2–S4 of the SI.

## Supporting Information Available

Experimental setup time-resolved in situ synchrotron X-ray diffraction experiments and Rietveld Refinement of selected samples (PDF)
