# Supplementary material for: Toward the Controlled Synthesis of Nanostructured Si and SiOx Anodes for Li-Ion Batteries via SiO2 Magnesiothermic Reduction Reaction
Source: ACS Appl Energy Mater. 2025 Feb 6;8(4):2249–59. doi: 10.1021/acsaem.4c02836 (PMC11863294; doi:10.1021/acsaem.4c02836)
Supplement: Supplementary file 1 — ae4c02836_si_001.pdf [file ae4c02836_si_001.pdf]

Supporting Information

Towards the controlled synthesis of nanostructured Si and SiO_x anodes for Li-ion batteries via SiO_2 magnesiothermic reduction reaction

Pedro Alonso Sánchez[a], Kesavan Thangaian[b], Ole Andreas Øie[b], Anders Gaarud[b], Miguel Rodríguez Gomez[a], Vadim Diadkin[c], Javier Campo[a], Federico Cova[d,*], María Valeria Blanco[a,*]

[a] Aragon Nanoscience and Materials Institute (CSIC - University of Zaragoza) and Physics Condensed Matter Department, C/Pedro Cerbuna 12, 50009 Zaragoza, Spain

[b]Department of Materials Science and Engineering, Norwegian University of Science and Technology, Trondheim 7491, Norway

[c] Swiss-Norwegian Beamline, European Synchrotron Radiation Facility, Avenue des Martyrs 71, 38042 Grenoble Cedex 9, France

[d] ALBA Synchrotron, Carrer de la Llum 2-26, Cerdanyola del Vallès 08290, Spain;

* E-mail: fcova@cells.es

* E-mail: mariavaleria.blanco@unizar.es

1. **Experimental setup used for time-resolved in situ synchrotron X-ray diffraction experiments.**

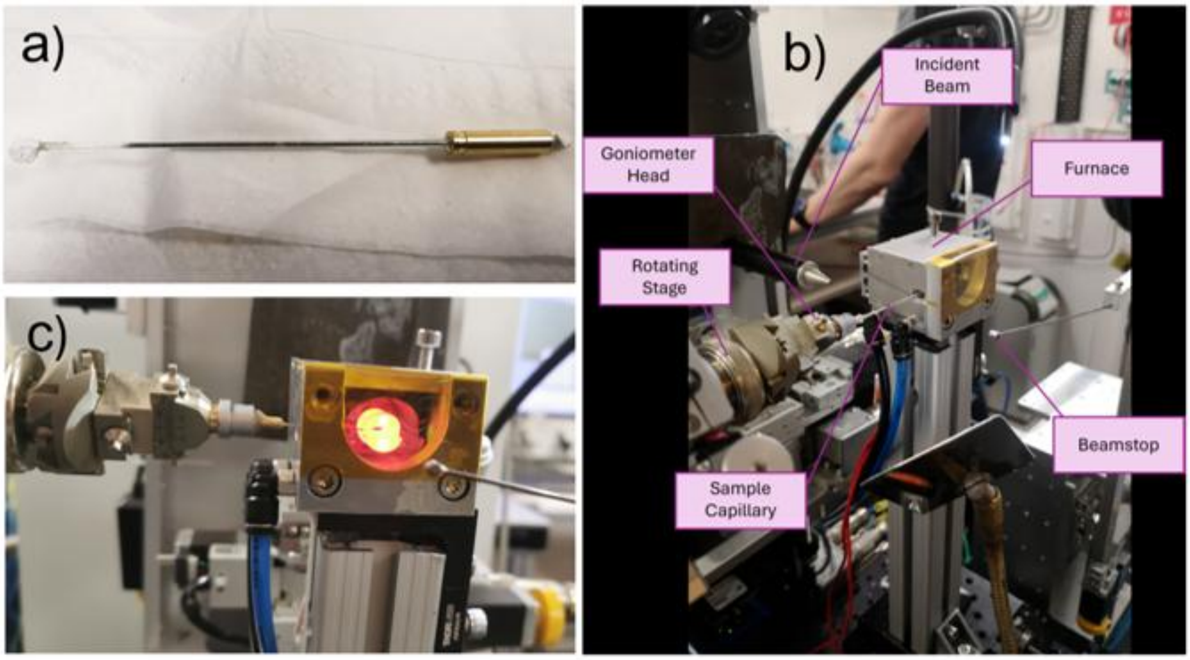

Fig. S1: a) Picture of sapphire capillary loaded with sample and sealed, b) Picture of experimental setup mounted at the BM01 Beamline indicating the different components, c) Picture of the experimental setup operated at high temperatures

2. Rietveld Refinement of selected samples

Rietveld refinement results from NZ-1-HT1 at selected temperatures are presented as to show the quality of the fitted data. Fig.S2, Fig.S3 and Fig.S4 display experimental and fitted data of diffraction patterns acquired when samples reached temperatures of 476ºC, 600ºC and 800ºC, respectively. Each figure contains the observed and calculated intensity and the corresponding identified phases. Bragg reflections from the spurious component located outside of the sample (mentioned in the main text) are also labelled.

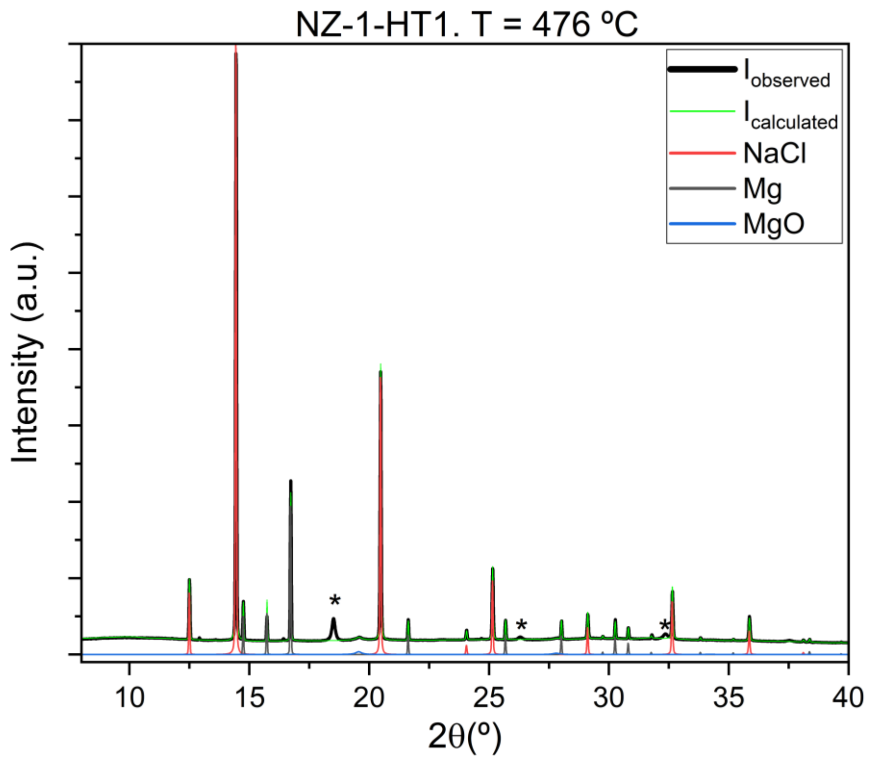

Fig. S2: Rietveld refinement at 476 ºC. Rwp = 12.3

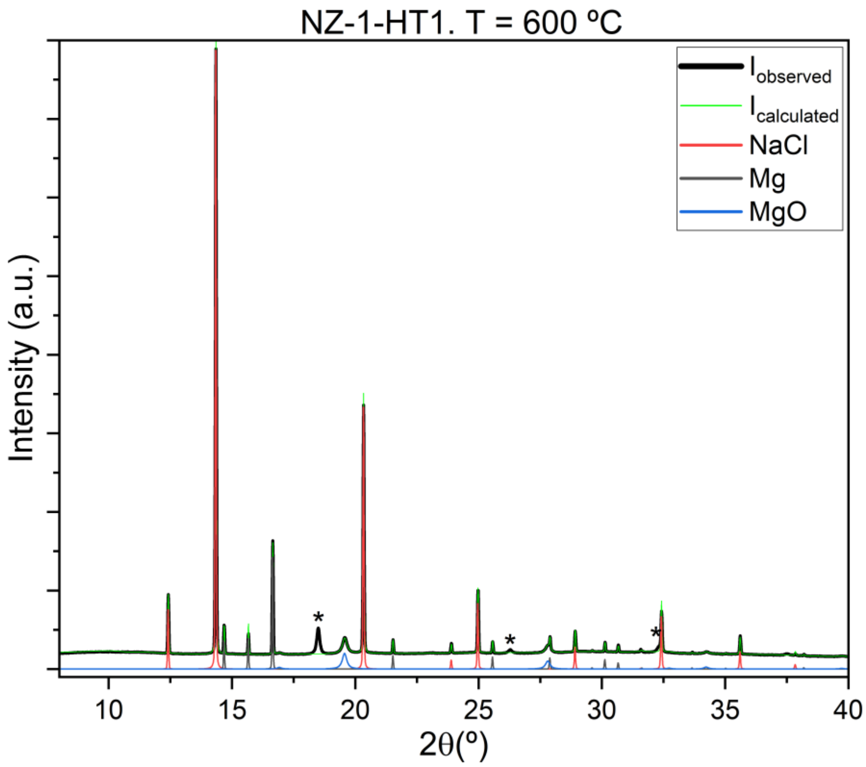

Fig. S3: Rietveld refinement at 600 ºC. Rwp = 10.6

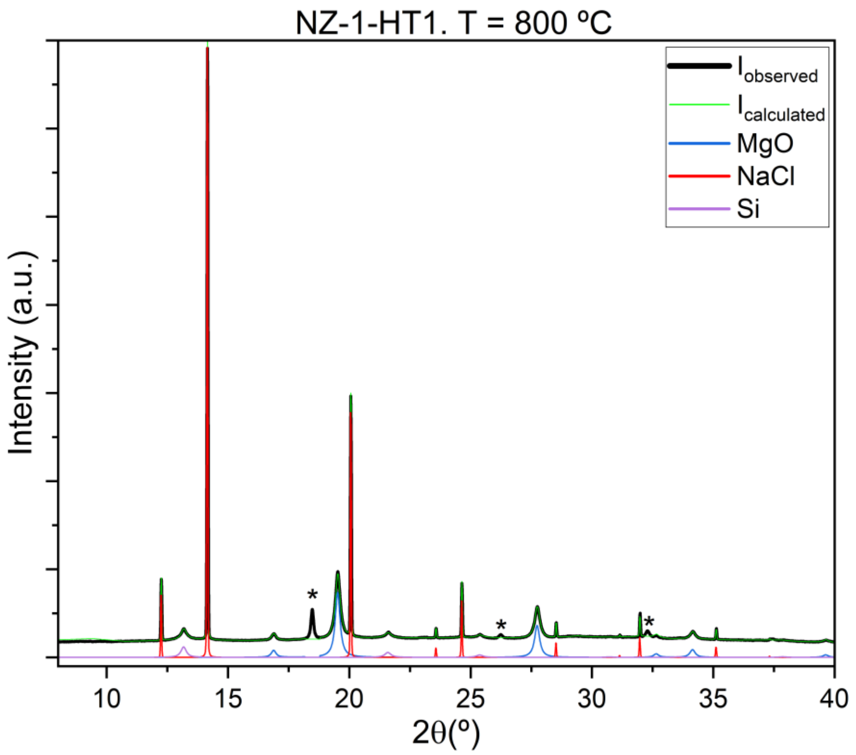

Fig. S4: Rietveld refinement at 800 ºC. Rwp = 10.12
